# Real-World Evidence of Langer’s Axillary Arch: Bridging Anatomical Variations and Surgical Practice for Safer Axillary Procedures

**DOI:** 10.7759/cureus.82177

**Published:** 2025-04-13

**Authors:** João Soares, Ricardo Vieira

**Affiliations:** 1 Dermatology, Unidade Local de Saúde de Coimbra, Coimbra, PRT

**Keywords:** anatomical variation, langer's axillary arch, lymphadenectomy, melanoma metastases, surgical anatomy

## Abstract

Langer’s axillary arch is an anatomical variant characterized by a fibromuscular band extending from the latissimus dorsi to the pectoralis major, humerus, or coracoid process, traversing the axillary region. This structure may alter expected anatomical relationships and has the potential to compress neurovascular elements, thereby complicating surgical procedures. Although extensively described in cadaveric studies, in vivo identification during surgery remains infrequently reported. Given its anatomical variability and potential impact on surgical outcomes, further intraoperative documentation of this variant is clinically relevant and may contribute to improved surgical safety and planning. We present the case of a 65-year-old female with left axillary lymph node metastases from melanoma, undergoing complete axillary lymphadenectomy. Intraoperatively, a fibromuscular structure consistent with Langer’s axillary arch was identified, extending from the latissimus dorsi and crossing the neurovascular bundle before inserting on the humerus. Careful dissection was performed to preserve vital structures and ensure oncologic completeness. The lymphadenectomy was successfully completed with histologically clear margins. The patient tolerated surgery well, received adjuvant immunotherapy, and remains disease-free after one year of follow-up. The recognition of Langer’s axillary arch during the procedure was critical in preventing inadvertent vascular or nerve injury, ensuring a safe and effective intervention. By bridging anatomical research with clinical practice, this case underscores the clinical importance of anatomy during surgical procedures in the axilla. Failure to recognize Langer’s axillary arch can increase the risk of surgical complications, including neurovascular injury or incomplete dissections. The *in vivo* documentation of this structure provides a valuable educational reference for surgeons, emphasizing the relevance of detailed anatomical understanding in improving patient outcomes.

## Introduction

The axilla is a complex anatomical region containing vital neurovascular structures and lymphatic tissue, often involved in dermatologic, oncological, reconstructive, and vascular surgeries such as lymphadenectomy for melanoma metastasis. While lymphadenectomy aims to achieve regional disease control and accurate staging, anatomical variations, such as the Langer's axillary arch, may complicate surgical approaches. This muscular or fibrous structure, which spans the axilla, can obscure landmarks, compress neurovascular bundles, or create unexpected challenges during dissection [1,2].

The Langer's axillary arch is a well-documented anatomical variation in cadaveric studies, with a reported prevalence ranging from 3% to 19% [3,4]. Karl Langer's original description of the axillary arch, termed "Achselbogen," was published in 1846 in the "Österreichische Medizinische Wochenschrift" [5,6]. This structure typically originates from the musculus latissimus dorsi and inserts variably on the humerus, musculus pectoralis major, or coracoid process [7]. Despite extensive anatomical descriptions in cadaveric studies, reports of its presence and implications during live surgical procedures are sparse, limiting our understanding of its clinical relevance and its potential impact on axillary lymphadenectomy [8-11].

This work aims to bridge the gap between anatomical research and clinical practice by presenting a case where Langer's axillary arch was identified and documented during lymphadenectomy for melanoma metastasis. By providing operative images and detailed descriptions, this study seeks to enhance the understanding of this anatomical variation, emphasize its importance in surgical planning, and contribute to better outcomes in procedures involving the axilla. These findings highlight the significance of integrating anatomical knowledge into surgical practice to manage challenging cases effectively.

## Case presentation

A 65-year-old female with a history of superficial spreading melanoma (Breslow thickness 0.7 mm, without ulceration) localized in the lateral region of the antebrachium sinistro was operated on two years prior and was under follow-up surveillance. She presented with matted lymph node metastases in the left subpectoral region. Fine needle aspiration cytology confirmed the diagnosis of melanoma metastasis. The patient was autonomous with no significant comorbidities. Staging was determined as T1aN1bM0, indicating the necessity for left axillary lymphadenectomy.

The lymphadenectomy involved levels I, II, and III of the axilla sinistra. During the procedure, critical anatomical structures were meticulously identified and preserved, including the nervus thoracicus longus, nervus thoracodorsalis, vena axillaris, arteria axillaris, and the plexus brachialis. The nervus thoracicus longus was traced along the musculus serratus anterior to avoid injury that could lead to scapula alata. The nervus thoracodorsalis, a key landmark innervating the musculus latissimus dorsi, was preserved to maintain humeral extension and adduction. The vena axillaris and arteria axillaris were carefully dissected, ensuring that vascular branches were not compromised. Additionally, the plexus brachialis, comprising major nerve trunks and cords traversing the axilla, was safeguarded to prevent functional deficits.

**Figure 1:**
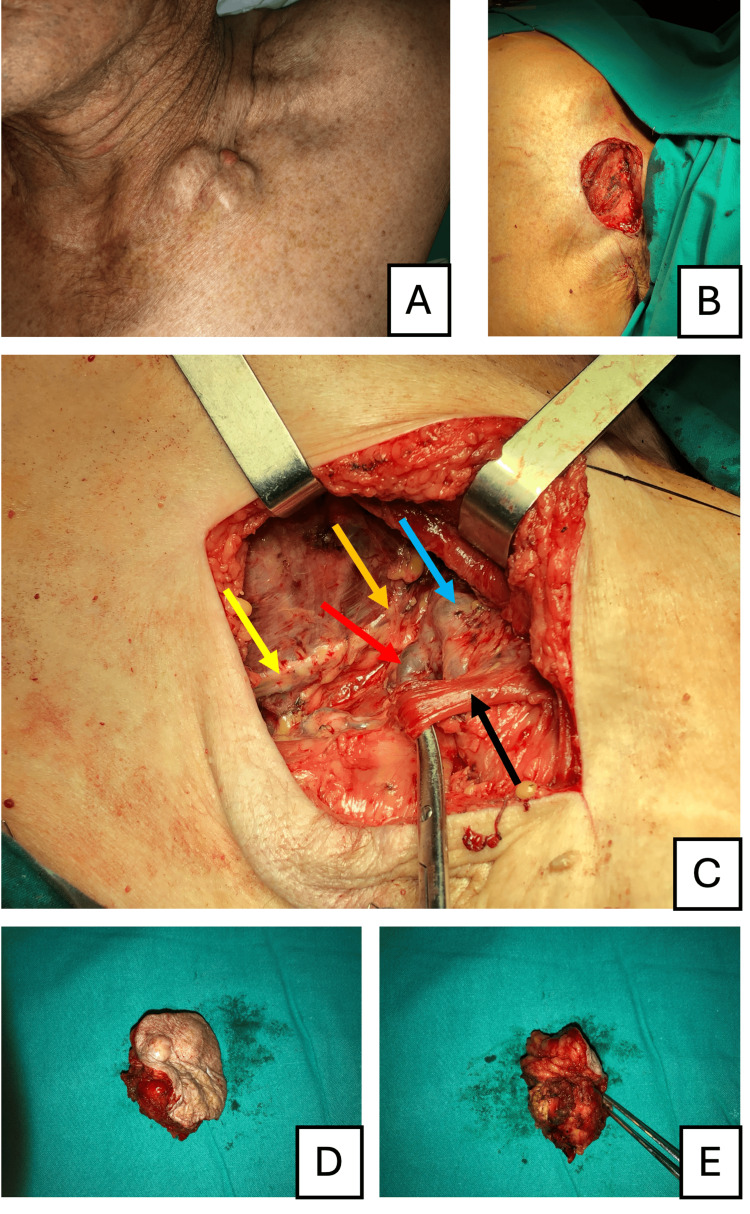
Preoperative and intraoperative findings in the management of axillary lymph node metastasis of melanoma (A) Preoperative image showing the matted lymph node metastases prominently visible in the subpectoral region. (B) Intraoperative view of the surgical defect after complete excision of the metastatic lymph node aggregate. (C) Identification of key anatomical structures during surgery, including the nervus thoracodorsalis (yellow arrow), the nervus thoracicus longus (orange arrow), the vena axillaris (blue arrow), the arteria axillaris (red arrow), and the Langer's axillary arch (black arrow). (D, E) Images of the excised matted lymph node metastases, confirming its dimensions and successful removal.

During dissection, a muscular variant consistent with the Langer's axillary arch was identified (Figure 1). This structure originated from the musculus latissimus dorsi, crossed the neurovascular bundle, and inserted proximally at the crista tuberculi majoris humeri, near the insertion of musculus pectoralis major.

The lymph node aggregate was completely excised with histologically clear margins, and there were no significant post-operative motor deficits. Postoperative treatment included immunotherapy with pembrolizumab, which was well-tolerated without significant toxicity. The patient remains disease-free with one year of follow-up.

## Discussion

The identification of the Langer's axillary arch, in this case, was critical in ensuring a successful outcome without complications. In this instance, the Langer's arch was found to cover the arteria axillaris, vena axillaris, plexus brachialis, and their branches, thereby obscuring key anatomical landmarks. Without the surgeon's anatomical awareness of this variation, the presence of the Langer's arch could lead to severe consequences, including neuromuscular compression, incomplete lymphadenectomy, or inadvertent injury to essential neurovascular structures [12].

Comparable clinical cases have also documented the axillary arch, most notably in breast and sentinel lymph node surgeries. For example, Keshtgar et al. [8] reported a case where the axillary arch influenced sentinel lymph node mapping, and Daniels and Querci della Rovere [7] highlighted its potential to complicate axillary clearance. In contrast, our study focuses on providing high-resolution color photographs and a detailed anatomical description, which may offer added educational value.

Additional imaging studies, such as ultrasound or MRI, could be useful preoperatively to identify anatomical variants. This is particularly relevant when a mass insinuates around the axillary vessels, making it less accessible through the arch and increasing the risk of complications. Preoperative identification of such variations could minimize surgical risks and significantly improve outcomes.

The educational value of this case lies in bridging the gap between descriptive anatomy and applied surgical anatomy with the in vivo identification of the Langer's arch during live surgery. This is particularly relevant for the ongoing training of surgeons, as awareness of this anatomical variation can enhance surgical techniques, reduce complications, and improve patient outcomes. Although cadaveric studies extensively describe Langer's arch, in vivo identification during live surgeries remains sparse [13]. The insights gained from this case may be valuable not only in dermatologic and oncologic surgeries, as exemplified here, but also in reconstructive and vascular procedures involving the axilla.

The limitations of this study include its nature as a single case, which restricts generalizations, especially given the inherent variability of human anatomy. Future research should focus on documenting similar cases, establishing the prevalence of the Langer's arch during live surgeries, exploring the potential role of preoperative imaging in routine surgical planning, and documenting potential complications related to this anatomical variation.

## Conclusions

Langer's axillary arch may complicate surgical approaches to the axilla, including dermatologic, oncologic, reconstructive, and vascular procedures. This case describes the in vivo identification of Langer's arch during live surgery, demonstrating the practical utility of anatomy in clinical practice. Awareness of this anatomical variation can prevent surgical injury, improve patient outcomes, and minimize surgical risks. Additionally, this case underscores the importance of integrating anatomical knowledge into surgical training and planning. Future research should aim to document similar cases and explore the role of advanced imaging techniques in preoperative assessment to enhance the safety and precision of axillary surgeries.
